# Detection of Human Leukocyte Antigen B27 by Flowcytometry in Patients With Suspected Ankylosing Spondylitis in a Tertiary Care Centre

**DOI:** 10.7759/cureus.13560

**Published:** 2021-02-25

**Authors:** Priyathersini N, Sri Gayathri Shanmugam, S. Sri Devi, Mohana Priya Chinambedu Dandapani, Rajendiran S, Lawrence D'Cruze

**Affiliations:** 1 Pathology, Sri Ramachandra Institute of Higher Education and Research, Chennai, IND; 2 Genetics, Sri Ramachandra Institute of Higher Education and Research, Chennai, IND

**Keywords:** hla b27, flow cytometry, indian population, direct immunofluorescence, rheumatoid factor, ankylosing spondylitis

## Abstract

Introduction

Human leukocyte antigen B27 (HLA-B27) is strongly implicated in the pathogenesis of ankylosing spondylitis (AS). Hence, HLA-B27 testing is routinely used in the diagnosis of AS.

Objectives

We aimed to establish the frequency of HLA-B27 in AS patients by flow cytometry and relate the differences between B27+ and B27- cases to the serum concentrations of rheumatoid arthritis factor (RA), erythrocyte sedimentation rate (ESR), and C-reactive protein (CRP).

Methods

The study population included a total of 210 patients who visited the tertiary health care center. The peripheral blood samples obtained from AS patients were subjected to a qualitative two-color direct immunofluorescence method using the HLA-B27/CD3 antibody for the rapid detection of HLA-B27 antigen expression in erythrocyte-lysed whole blood in FACSCalibur flow cytometer (Becton Dickinson, USA).

Results

Out of 210 AS patients, the distribution of HLA-B27 positivity was observed only in 46 (22%) patients. The remaining 164 patients (78.1%) were negative for HLA-B27. Of the 46 HLA positive patients, 39 (25.34%) were males and only seven (12.5%) were females. In both sexes, HLA-B27 frequency was significantly higher in the age group 21-30 years, followed by 41-50 years. The current study also revealed a significant association between sex and age of onset of HLA‑B27 detection in patients with suspected AS. Disease activity was not significantly correlated with RA, ESR, and CRP.

Conclusions

The detection of HLA-B27 by flow cytometry proved to be a reliable test in the screening of AS in the Indian population.

## Introduction

Ankylosing spondylitis (AS), one of the most frequent forms of spondyloarthritis (SpA), predominantly affects the axial musculoskeletal system. The primary causes of the disease revealed sacroiliitis and spondylitis, with the formation of syndesmophytes leading to ankylosis and eventual loss of spinal mobility [1,2]. Peripheral arthritis, inflammatory back pain, enthesopathy, and asymmetrical peripheral oligoarthritis have also been observed in AS patients [2]. The disease can also be accompanied by extraskeletal manifestations, such as anterior uveitis, psoriasis, chronic inflammatory bowel disease, cardiovascular or pulmonary complications [2-4]. The incidence of AS varies from 0.5 to 14 per 100,000 people per year, and the prevalence of the disease is low, affecting 0.1 and 1.0% of general populations [3]. AS is an autoimmune disease with >90% of the risk of developing the disease is found to be genetically determined [5,6]. A strong genetic predisposition was confirmed by the discovery of a remarkably high association between AS and HLA-B27 in 1973 [7]. Even though HLA-B27 is present in more than 90% of patients in the majority of ethnic groups that present ankylosing spondylitis, a proportion of AS cases that do not involve HLA-B27 have also been reported [8].

HLA-B27 is a major histocompatibility complex (MHC) class I molecule, which is a cell-surface glycoprotein and expressed on antigen-presenting cells. The presence of HLA-B27 in the MHC locus contributes to ~20.1% of AS, while 4.3% is found in loci other than HLA-B27 [6,9]. HLA-B27 is a unique human leukocyte antigen (HLA) class I molecule, as it has a characteristic amino acid composition from other class I molecules and is found only in AS [10]. The presence of the HLA-B27 antigen is strongly associated with AS and a few other rheumatic disorders (Reiter’s syndrome, acute anterior uveitis, and inflammatory bowel disease [3,4]. HLA-B27 testing is routinely used to diagnose AS since the HLA-B27 surface antigen is expressed in 90% of AS patients as compared to 8% of healthy individuals [11]. The risk of developing AS is substantially higher in HLA-B27-positive relatives as compared to HLA-B27-positive cases [12]. Around 100 HLA-B27 subtypes have been identified. HLA-B*2705 is the most prevalent subtype, present in almost every population in the world and considered to be the “parent” HLA-B27 molecule, while B*2702, B*2704, and B*2707 have evolved from the parent molecule and are the other subtypes found in AS patients [13,14]. These subtypes differ in their amino acid composition, which alters the peptide-binding properties of the molecule [10].

The MHC class I molecules are heterotrimers, composed of a heavy chain, β2 microglobulin (β2m) light chain, and an oligopeptide. These peptides are generated in the cytosol, processed in proteosomes, and then assembled in the endoplasmic reticulum (ER). The oligopeptide folds into three domains (α1, α2, and α3) and along with β2m forms an extracellular domain. The domains α1 and α2 form the peptide-binding site for antigenic peptides of 8 to 10 amino acids in length [15]. ER aminopeptidases (ERAP1) cleave the precursors to oligopeptides of eight or nine residues in length, which is optimal for binding to HLA-B27. These peptide MHC complexes will undergo glycosylation and tertiary folding in the Golgi apparatus to generate mature epitopes [16]. Subsequently, HLA-B27 heavy chains form heterotrimeric complexes with 2-microglobulin (2m) and intracellular peptides derived from self-proteins, viruses, and bacteria. In the absence of β2m, heavy chains will misfold, and ER-associated degradation may occur in the ER. Several theories have been proposed to explain the molecular pathogenic role of HLA-B27 in AS. The onset of AS may result from aberrant peptide processing and presentation, misfolding of HLA-B27 molecules, the formation of heavy chain homodimers [17], or β2m accumulation and deposition. These abnormalities become the target of autoreactive CD8+ T cells, which cause cytotoxicity resulting in chronic inflammation [16].

HLA-B27 can be detected by a variety of methods with similar sensitivity and specificity, including the classical complement-dependent cytotoxicity (CDC) and DNA-based genotyping methods. However, both these methods are expensive and time-consuming as compared to flow cytometry. Besides, the CDC method is based on sera, generating cross-reactivities. As a consequence, several companies developed various anti-HLA-B27 monoclonal antibodies to be used in flow cytometry. Hence, in recent years, HLA-B27 detection in peripheral blood lymphocytes using the HLA-B27 monoclonal antibody by flow cytometric technique is widely adopted in diagnostic laboratories. Further, as compared to other techniques, this method is simple, cost-effective, and takes a short turnaround time.

Although there is mounting evidence on HLA-B27 worldwide, most of the studies were devoted to genetic studies; only a few reports are available with respect to the role of flow cytometry in the detection of HLA-B27. Flow cytometry is the most widely used diagnostic method for the detection of HLA-B27. Further, the studies related to the HLA-B27 association with AS in the Indian population are very few, and earlier research reported a broad range of frequency from 18% to 94% [17]. Hence, the present investigation was undertaken to study the frequency and the prevalence of HLA-B27 among AS patients in the Indian population in a tertiary care center. The second objective of the study is to relate the differences between B27+ and B27- cases to the serum concentrations of rheumatoid arthritis factor (RA), erythrocyte sedimentation rate (ESR), and C-reactive protein (CRP). RA, ESR, and CRP are the currently used biomarkers for evaluating the inflammatory activity of the disease.

## Materials and methods

Study population

This prospective study was conducted on the patients reporting to the Rheumatology, Orthopaedics, General Medicine, and General Surgery clinics at the Sri Ramachandra Medical College, Chennai, India. The study was carried out between January 2015 to March 2018. The study protocol was duly approved by the Institutional Ethical Committee in Sri Ramachandra Institute of Higher Education and Research (Ethics Committee no: CSP-MED/19/MAR/51/36), and all patients gave written informed consent for participation. Consecutive sampling method was used. Inclusion criteria: patients who fulfilled the Assessment of SpondyloArthritis International Society (ASAS) criteria [18] for AS were considered for the study. Exclusion criteria: patients who were not willing to participate in the study and patients with inadequate data were excluded. The study population comprised 210 AS patients (56 females, 154 males) between the ages of five and 65 years. A detailed medical history was obtained from each patient, and all study participants were thoroughly taken for a physical examination. After obtaining their demographic data (name, age, gender, and address), all the selected patients were examined by the physicians of the concerned departments for evaluating the necessary radiographs, and clinical tests were performed to confirm the clinical status. All patients with AS were unrelated. All patients were also tested for complete blood count (CBC) by a semi-automatic hematology counter. Serum CRP levels were measured by the immunonephelometric method (Siemens, Munich, Germany) according to the manufacturer’s instructions, and results were expressed as mg/L. ESR was measured by capillary photometry, and results were expressed as mm/hr. C-reactive protein estimation by semi-quantitative latex agglutination method in addition to HLA-B27. Control subjects, who were free of any history of rheumatic diseases and no family history of AS, were selected from a healthy group. A total of 210 AS patients who were willing to participate and with adequate data for inclusion were selected for the study.

Daily instrument setup and quality check (QC)

The BD FACS Calibur flow cytometer is equipped with an air-cooled argon laser excitable at 488 nm and a diode laser emitting red light at 640 nm with four fixed fluorescence detectors [533/30 nm (FL1), 585/40 nm (FL2), >670 nm Long Pass (FL3), and 675/25 nm (FL4)]. When the fluorochrome-conjugated cells intercept the laser beam in the flow cell, scattered and fluorescent light provide information about particle size, shape, granularity, and fluorescence intensity. For quality control of optics, electronics, and fluidics of the system, a “Pass” on the Instrument QC report had to be obtained. To accomplish this, BD FACSComp^TM^ software was run on the BD FACSCalibur instrument using BD Calibrite^TM^ beads to optimize instrument settings. These beads optimize photomultiplier tube (PMT) voltage settings, fluorescence compensation, and sensitivity. Subsequently, the HLA-B27 assay setup was performed using BD^TM^ HLA-B27 calibration beads to set fluorescein isothiocyanate (FITC)/FL1 detector voltage specifically for the assay by entering the bead suffix value correctly in the BD FACSComp^TM^ software to set the decision-maker [2,19]. The suffix is found on the HLA-B27 reagent vial and is represented as units of log median fluorescence (LMF). The software then adjusts the detector voltage until the bead attains the target value. Both BD FACSComp and BD HLA-B27 QC were performed every day before a clinical diagnostic sample was tested [2].

Blood samples

Peripheral blood (3 ml) was collected aseptically by venipuncture, using ethylenediaminetetraacetic acid (EDTA) vacutainers from patients with suspected AS.

HLA-B27 determination

Peripheral blood was used to analyze the expression of HLA-B27 antigen on the T cell surface using a monoclonal antibody specific for HLA-B27 (HLA-B27 Kit, Becton Dickinson, San Jose, CA, USA) by flow cytometry technique. Blood samples were stained with anti-HLA-B27 antibody conjugated with fluorescein and with anti-CD3 antibody conjugated with phycoerythrin for 20 min in the dark. When anti-HLA-B27 FITC/CD3 PE monoclonal antibody reagent was added to human whole blood, the fluorochrome-labeled antibodies are bound specifically to the leucocyte surface antigens.

Sample processing

The sample was mixed by tilting it upside down. The whole procedure was performed following the instructions recommended by the manufacturer. Fifty microlitres of blood samples were taken in a 12 X 15 mm BD Falcon tube, and 30 µl of the antibody to HLA-B27 was added, vortexed at low speed for about 2-3 seconds, and incubated for 20 min in the dark. The stained samples were treated with 2 ml of 1X BD FACS^TM^ lysing solution (200 µl of 10X lysis buffer was made up to 2000 µl with distilled water) for 10 min to lyse the erythrocytes. After adding the lysing solution, the sample was vortexed at low speed for about three seconds and incubated for 15 minutes, and centrifuged at 500 X g for 5 min at room temperature. The supernatant was aspirated, leaving 50 µl of residual fluid. Two milliliters of 1X phosphate-buffered saline (PBS) with 0.1% sodium azide was added to the residual fluid, vortexed at low speed, and centrifuged at 500 X g for 5 min again. The previous step was again repeated by aspirating the supernatant and leaving 50 µl of residual fluid. To this, 450 µl of 1X PBS was added, vortexed at low speed, and the samples were acquired for 15,000 events in BD FACSCalibur using HLA-B27 v3.1 software (Becton Dickinson, San Jose, CA, USA) [2]. If the sample had to be stored, 0.25 µl of 1% paraformaldehyde (PFA) in PBS was added.

Sample analysis

The acquired samples were then be analyzed by HLA-B27 acquisition software (v3.1). When around 15,000 total events or 2,000 CD3 T lymphocytes were acquired, a dot plot of PE (FL2 detector) versus side scatter (SSC) was used by the software algorithm to gate the CD3-positive fluorescent cells. The LMF intensity of the HLA-B27 FITC signal (FL1) was calculated for the CD3 T lymphocytes and compared to the predetermined decision marker. The T-lymphocyte population was displayed in a FITC/FL1 histogram, where the LMF was calculated. Samples were considered as HLA-B27-positive if the LMF was greater than or equal to the decision-maker. Samples with an LMF less than the decision marker were considered as HLA-B27-negative.

Statistics

The demographic, clinical, and technical data were collected using a ‘data collection form’ and entered into a computerized database before statistical analysis. The accrued data was then analyzed using Statistical Package for Social Sciences (SPSS) software (IBM SPSS Statistics, Armonk, NY). As it was a case-control study, only the odds ratio (OR) and p-values were calculated. The p-value of less than 0.05 was considered statistically significant.

## Results

Clinical characteristics

Age and Sex Distribution in AS

**Figure 1:**
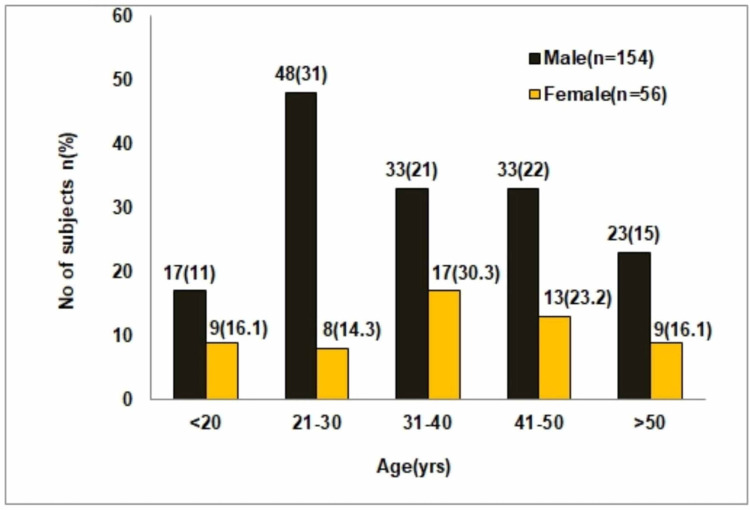
Age and sex distribution of study population

This study was a prospective hospital-based study comprising of 210 AS patients, who were matched to gender with healthy controls. Figure 1 reveals the demographic data of patients in various age groups (<20 years, 21-30, 31-40, 41-50, and >50 years), ranging between 12 to 60 years. In males, the peak incidence was in the age range of 21-30 years (31%), followed by 41-50 years (22%) with a mean age of 36.5 years. There was no significant difference between 31-40 (30%) and 41-50 (23%) years. In females, the maximum number of AS patients was within 31-40 (30%) years, followed by 41-50 (23%) years.

Among the study group of 210 AS patients, 56 (27%) were females and 154 (73%) were males, representing a ratio of 0.7:1.85. Out of 210 AS patients, the distribution of HLA-B27 positivity was observed only in 46 (22%) patients (Table 1).

**Table 1:** HLA-B27 frequency in the study population

HLA-B27 status	Frequency
HLA Positive (Both male and female)	46 (21.90%)
HLA Negative (Both male and female)	164 (78.10%)
Total	210

**Figure 2:**
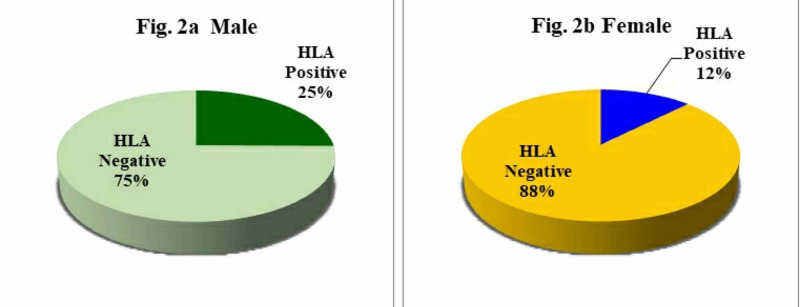
Frequency of HLA-B27 in the suspected AS patients according to gender

The remaining 164 patients (78%) were negative for HLA-B27 antigen. Of the 46 HLA-positive patients, 39 (25.34%) were males and only seven (12.5%) were females (Figures 2a, 2b). 

**Figure 3:**
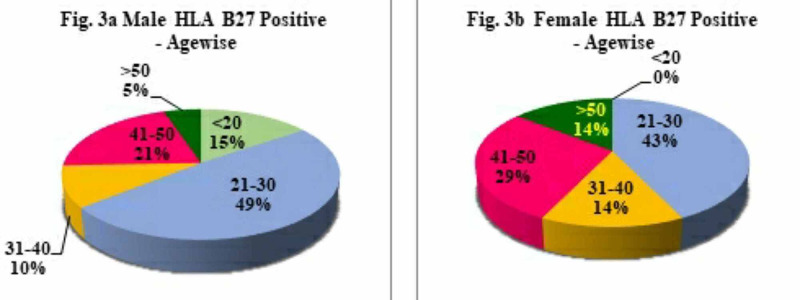
Distribution of HLA-B27 among different age groups in the study population

Among the different age groups of patients, the frequency of HLA-B27 positivity showed a similar trend in both males and females. In males, the frequency was found to be 15.4%, 48.7%, 10.3%, 20.5%, and 5.13% in the <20 years, 21-30, 31-40, 41-50 and >51 years age groups, respectively (Figure 3a). In females, 0%, 42.86%, 14.29%, 28.57%, 14.29% in the <20 years, 21-30, 31-40, 41-50 and >51 years age groups, respectively (Figure 3b). In both sexes, HLA-B27 frequency was significantly higher in the age group 21-30 years (48.7%, 42.9%, respectively), followed by 41-50 years (20.5%, 28.6%, respectively) (Figures 3a, 3b). HLA-B27 positivity showed no significant difference in both younger (<20 years) or older (>50 years) age groups in both males and females (Figures 3a, 3b).

RA, ESR, and CRP in AS

To determine whether disease activity and B27 status were associated with serum concentrations of RA, ESR, and CRP in AS patients, these three parameters were measured and evaluated as inflammatory markers of AS. To accomplish this, AS patients were classified as B27 positive cases and B27 negative cases. Each category was analyzed to establish whether there was any significant correlation between B27 positive and negative cases to each of the serum proteins. Out of 86 patients in whom RA was tested, 21 patients (24%) were positive for HLA-B27, and the remaining 65 patients (76%) were negative for HLA-B27. All the HLA-B27 positive cases were RA negative (Table 2).

**Table 2:** RA in HLA-B27 positive and negative patients RA: Rheumatoid Arthritis Factor

Total Number of patients for RA = 86			
HLA-B27 Positive		HLA-B27 Negative	
21 (24.4%)		65 (75.5%)	
RA Positive	RA Negative	RA Positive	RA Negative
--	21	1 (1.5%)	64 (98.4%)

Both RA and AS can be similarly characterized by the presenting symptoms, radiographic characteristics, and serological testing. However, the diagnosis can be a challenge, and more tests may be required to establish the correct etiology. RA was checked to rule out seropositive arthropathy. Hence, RA was included in the study. In the B27 negative phenotypes, only one patient (2%) was positive for RA value. 

Out of 124 cases in which serum CRP concentrations were recorded, 30 patients (24%) were HLA-B27 positive, while the remaining 94 patients (76%) were B27 negative. Of the 30 B27 positive cases, 15 patients (50%) were CRP positive, and the remaining 50% were CRP negative. Of the 94 B27 negative phenotypes, only in 18 patients (19%), CRP levels were elevated, while in 76 patients (81%), CRP levels remained low (Table 3). 

**Table 3:** CRP in HLA-B27 positive and negative patients CRP: C-reactive Protein

Total Number of patients for CRP = 124			
HLA-B27 Positive		HLA-B27 Negative	
30 (24.1%)		94 (75.8%)	
CRP Positive	CRP Negative	CRP Positive	CRP Negative
15 (50%)	15 (50%)	18 (19.1%)	76 (80.8%)

Out of 154 cases, only 37 patients (24%) were positive for HLA-B27, the remaining 117 patients (76%) were B27 negative. In the 37 B27 positive phenotypes, ESR concentrations were elevated only in 18 patients (49%), while the remaining 19 patients (51%) were ESR negative. In the HLA negative phenotypes, 46 patients (39%) were ESR positive, and the remaining 71 patients (61%) were ESR negative (Table 4). 

**Table 4:** ESR in HLA-B27 positive and negative patients ESR: Erythrocyte Sedimentation Rate

Total Number of patients for ESR = 154			
HLA-B27 Positive		HLA-B27 Negative	
37 (24.02%)		117 (75.95%)	
ESR Positive	ESR Negative	ESR Positive	ESR Negative
18 (48.64%)	19 (51.35%)	46 (39.31%)	71 (60.69%)

The association of the biomarkers CRP and ESR with HLA-B27 was analyzed using Spearman’s rank correlation, and a significant correlation was noted in ESR (Table 5).

**Table 5:** Statistical analysis of HLA-B27 correlation with inflammatory markers CRP: C-reactive Protein, ESR: Erythrocyte Sedimentation Rate *p-value<0.05 considered as significant

Variables	Spearman’s rank correlation	P-value
CRP	0.05	0.4
ESR	0.15	0.02*

## Discussion

This prospective case-control study, first of its kind, was carried out to evaluate the association of HLA-B27 in patients with suspected AS by flow cytometry in a tertiary care center. Besides, different clinical parameters were assessed in males and females at various age groups (<20, 21-30, 31-40, 41-49, and > 51 years). Apart from AS, HLA-B27 is also associated with other rheumatic disorders, collectively known as SpA, that includes psoriatic arthritis, reactive arthritis and arthritis with inflammatory bowel disease, Reiter’s syndrome, and acute anterior uveitis [3,4]. The degree of association between HLA-B27 and SpA varies markedly among different diseases of this group and also between different populations [20]. 

In the present study, a total of 210 patients were enrolled, including 154 males (73.3%) and 56 females (26.7%) with maximum numbers of patients within 21 to 30 years (48.7%, 42.9%), followed by 41-50 years (20.5%; 28.9%) of age in both males and females. HLA-B27 positivity decreased with the increase of age, and at >51 years of age group, it decreased to 5.10% in males and 14.3 in females. In the present study, the youngest patient was five years old, and the oldest was 73 years with a maximum age range of 20-30 years. The present study demonstrated a significant association between sex and age of onset of HLA-B27 detection in AS patients. The present study also revealed 21.9% overall positivity for HLA-B27 with a significantly higher prevalence of HLA-B27 among male patients than females [20]. A number of studies have shown that men constituted three to four-fold higher number of patients than women in AS and had a significantly lower mean age at diagnosis in males. In the present study, male preponderance (25.3%) was observed as compared to females (12.5%), representing a ratio of 0.88: 2.41, thereby suggesting that males are more prone to AS than females in the Indian population. This is consistent with a recent study by Haridas et al. (2018) [21], who have shown that males were more frequently affected than females (male: female ratio of 3.4:1) in the Indian population. 

The flow cytometry analysis of HLA-B27 undertaken in the current study confirms the well-established association of AS with HLA-B27 in the Indian population, occurring in the frequency of 22%. In contrast, other studies have shown a higher frequency of HLA-B27 positivity in the Indian population with AS. In another study, when seronegative SpA patients who were negative for RA factor were tested, HLA-B27 frequency was more prevalent in children (68.75%) and males (81.81%) [11]. Thomas et al. (2006) reported that the frequency of HLA-B27 was 46% in SpA patients [22]. Another study has shown a 56% B27 frequency from patients of Asian Indian origin, with male predominance [17]. This may be due to the variation in the methodology adopted to detect HLA-B27. Flow cytometry assay can give false-negative results due to the cross-reactivity of GS145.2 antibody with HLA-B27 [14]. However, in recent years, flow cytometry is being increasingly appreciated as the gold standard method for the diagnosis of AS and other related diseases, as this method cost-effective, simple with a high degree of sensitivity, quicker reporting of test results, better testing output, for its ease of use and suitability of automation.

ESR and CRP tests are often done to detect or monitor patients with suspected inflammatory disorders. CRP is one of the acute phase reactants whose blood levels increase in response to inflammation. Increased levels of CRP generally are reflective of underlying inflammation, such as that resulting from trauma or infection. In contrast, deceptively low CRP levels may be found in patients with infections caused by low virulence organisms or in those treated with antibiotics. The reference ranges for normal RA level was < 20. The values of ESR > 15 mm/hr and CRP > 1.0 mg/dL were determined to be abnormal. In the present study, the clinical evaluation of the inflammatory markers in AS patients was less specific with respect to HLA-B27. All the B27 positive phenotypes were negative for RA, indicating no specific relationship between RA value and HLA-B27 positivity. This finding is consistent with a previous report, which has shown that AS patients were RA negative [22,23]. The other two inflammatory markers, CRP and ESR, were elevated only in 50% of the HLA-B27 positive phenotypes. Besides, HLA negative cases gad a higher percentage of ESR abnormality (39%) than CRP (19%). Therefore, from the present findings, it is inferred that ESR and CRP levels do not correlate strongly with HLA-B27 positive phenotypes of AS disease activity measures. A number of methods are available for HLA-B27 detection. Single nucleotide polymorphism (SNP) method had 78.6% sensitivity and 98.7% specificity. Screening with exon 2 (5' fragment) and exon 3 reverse transcriptase-polymerase chain reaction (RT-PCR) provided 100% sensitivity. Further testing with exon 2a and 2b fluorescence resonance energy transfer (FRET) RT-PCR produced 100% specificity [24].

Early diagnosis of SpA is important in the screening of other diseases like anterior uveitis, pulmonary consolidation, renal diseases, etc., in which HLA-B27 will be a predisposing factor. It is particularly more helpful in the diagnosis of SpA in young individuals before the appearance of radiological changes. HLA-B27 detection is also useful for screening of relatives who may have HLA-B27 antigen and at risk of developing SpA. Even though the current study is able to reflect the HLA-B27 status in AS patients of the Indian population, it has a limitation of a small sample size. Therefore, further extensive studies with a higher number of suspected AS patients are required to confirm the prevalence of HLA-B27 in AS patients in the Indian population.

## Conclusions

Taken together, the present study established the detection of HLA-B27 in AS patients of the South Indian population by flow cytometry technique, which proves to be a reliable, inexpensive screening test with high specificity and sensitivity and can be safely used by the clinicians. The sensitivity of the HLA-B27 screening test by flow cytometry was 99%, while the specificity was 100%. To the best of our knowledge, the present study is the first of its kind to assess HLA-B27 status by flow cytometry among the Indian population. In AS, since HLA-B27 association with axial manifestation was confirmed, HLA-B27 testing is routinely applied in the diagnosis of this disease. It may provide a clue to diagnosis before the onset of radiological changes, and also it may help in identifying at-risk family members.
